# Value of perioperative genitourinary screening culture and colonization status in predicting early urinary tract infection after renal transplantation

**DOI:** 10.1371/journal.pone.0196115

**Published:** 2018-04-19

**Authors:** Ahram Han, Sanghyun Ahn, Seung-Kee Min, Jongwon Ha, Yon Su Kim, Curie Ahn, Sang-il Min

**Affiliations:** 1 Department of Surgery, Seoul National University College of Medicine, Seoul, Korea; 2 Department of Internal Medicine, Seoul National University College of Medicine, Seoul, Korea; Medical University of Gdansk, POLAND

## Abstract

**Background:**

We aimed to assess whether patients colonized with certain organisms in the genitourinary tract would have greater urinary tract infection (UTI) risk during the post-transplantation period, and whether information on the perioperatively colonized organisms may help identify the causal organisms during early UTI.

**Methods:**

We retrospectively reviewed the culture results of preoperative urine, preoperative urethral swab, and postoperative urinary catheter tip specimens of 420 renal transplant recipients. The colonization status was compared to the culture results during the first UTI episode within 6 months after transplantation.

**Results:**

Twenty six (6.2%) patients developed early UTI, and the presence of common uropathogens in the perioperative genitourinary specimen was positively associated with a higher early UTI risk odds ratio [OR], 3.23; 95% confidence interval [CI], 1.44 to 7.24; *P* = 0.003). However, the actual causal organism during UTI was observed perioperatively only in 15 patients (40.5%). Neither perioperative colonization nor early UTI was associated with subsequent acute cellular rejection or graft failure.

**Conclusions:**

Renal transplantation patients who were colonized with common uropathogens were more likely to develop early UTI. However, the usefulness of the culture results of perioperative colonizers in predicting the causal organism during early UTI seems limited due to the low concordance rate.

## Introduction

Urinary tract infection (UTI) is the most common infectious complication following renal transplantation. The reported incidence rates vary widely among previous studies due to the differences in the UTI definitions and study periods [1, 2]. Adequate management of UTI is particularly important in kidney recipients, as it is known to be associated with an increased incidence of rejection, higher patient morbidity, and increased medical costs [3–8].

All symptomatic UTIs after renal transplantation are considered to be complicated UTIs [1]. The current guidelines recommend that all cases of symptomatic UTIs after transplantation should be treated with antibiotics, particularly with intravenous antibiotics after admission in cases of upper UTI [9, 10]. The management of patients who are initially treated empirically with antibiotics is subsequently adjusted based on the causative organism identified [10]. The choice of antibiotics administered during empirical therapy is guided by the local prevalence of pathogens, circumstances of infection (nosocomial or community-acquired), and the causative organisms isolated during the previous UTI episodes [10]. The recent emergence of multi-drug resistant organisms has further complicated the optimal initial management of UTI. The use of broad-spectrum antibiotics may induce microbial resistance, although inadequate initial coverage could also lead to the progression of infection [11, 12]. Due to the long duration required to obtain positive culture results, attempts have been made to predict the causal organisms using information obtained from surveillance cultures prior to the event in ventilator-associated pneumonia [13–15] and critically ill patients with sepsis [16, 17]. However, such approaches have not been used in cases of UTI following solid organ transplantation.

The 2 major routes through which bacterial pathogens enter the urinary tract include ascending migration from the perineum and hematogenous spread. The development of genitourinary colonization or UTI by the entering organism is determined by several host factors, including behavioral, environmental, and immunologic factors, as well as the virulence of the invading organism [18]. During the early post-transplantation period, when the immune function of recipients is impaired, colonized organisms may be an important source of infection [19].

In the present study, we hypothesized that patients colonized with certain organisms in the genitourinary tract, will have a greater risk of UTI during the early post-transplantation period, and that the information on the perioperatively colonizing organisms could help identify the causal organisms in cases of UTI. To test this hypothesis, we retrospectively reviewed the culture results of perioperative genitourinary specimens and assessed their association with the development of early UTI and subsequent causative organisms isolated during early UTI episodes.

## Materials and methods

### Study design and participants

Patients who received renal allograft between July 2005 and June 2010 at Seoul National University Hospital, Korea were included in this study. Patients who had undergone simultaneous transplantation of other solid organs, those with renal graft survival for <2 weeks, and those who were lost to follow-up within 5 years were excluded. The study was reviewed and approved by the Institutional Review Board of Seoul National University Hospital (IRB number: 1506-111-682). Documentation of written informed consent was waived by the Institutional Review Board, as all data were fully anonymized before analysis.

### Perioperative genitourinary surveillance cultures

In patients undergoing renal transplantation, 3 types of genitourinary surveillance culture specimens were obtained perioperatively: preoperative midstream urine, preoperative genitourinary swab, and postoperative urinary catheter tip. The initial voided midstream urinary specimen and genitourinary swab specimen were collected within 2 days prior to transplantation. In patients who were not able to submit preoperative midstream urine samples, urine samples were collected in the operating room after aseptic insertion of the urinary catheter. In patients who had less than 20cc of urine in the bladder at the time of catheter insertion, the bladder was irrigated with 50 cc of normal saline, and the irrigated fluid was cultured instead. The urinary catheter tip was cultured following its removal, usually on the third postoperative day.

### UTIs

Bacteriuria was defined as bacterial growth of ≥10^5^ colony forming units per milliliter (cfu/mL). UTI was defined as the presence of bacteriuria along with one of the following symptoms: dysuria, urgency, frequent voiding, residual urinary symptoms, flank pain, or fever. UTI was further classified as pyelonephritis (UTI with fever, along with chills, flank pain, or graft area tenderness on physical examination) and cystitis (symptomatic UTI that did not meet the pyelonephritis criteria). Early UTI was defined as UTI within 6 months posttransplantation.

When comparing the microorganisms isolated during the UTI episodes and the microorganisms isolated from the perioperative surveillance cultures, microorganisms were considered identical if they were identical in terms of species and antibiotic susceptibility profile. UTIs by multidrug-resistant (MDR) bacteria were defined as those by organisms with non-susceptibility to at least 1 agent in ≥3 antimicrobial categories; intrinsic resistance was not considered [20]. An uropathogen was defined as an organism known to be clearly associated with UTI, including *Escherichia coli*, and *Klebsiella*, *Enterobacter*, *Pseudomonas*, and *Enterococcus* species.

### Transplant protocols: Immunosuppression protocol and antibiotic prophylaxis

During the study period, the induction regimen of the recipients was determined based on the immunological risks. Recipients who received a kidney from first-degree living related donors did not receive any induction therapy, whereas most other recipients received basiliximab. The initial maintenance therapy included steroids, mycophenolic acid, and tacrolimus or cyclosporine. After an intraoperative single dose of methylprednisolone was administered, the steroid dose was rapidly reduced from 1 mg/kg/day methylprednisolone to 5 mg/day oral prednisone within one month. Tacrolimus treatment was initiated at 0.075 mg/kg, twice a day, and was adjusted to achieve serum target trough concentrations of 8–12 ng/ml for up to 3 months, 6–8 ng/ml until 12 months, and 4–6 ng/ml thereafter. The dose of immunosuppressive agents was changed or reduced in cases with drug-related adverse events.

All patients received perioperative antibiotic prophylaxis comprising 1 g of intravenous cefazolin administered at 8-hour intervals for 3 days, regardless of the perioperative surveillance culture results. The first dose was administered on the day of the surgery, within 1 hour prior to incision.

At our institution, postoperative trimethoprim-sulfamethoxazole (TMP-SMX) prophylaxis for *Pneumocystis jiroveci* was not a common practice during the study period, and was administered only in 17 patients (4.0%). Cytomegalovirus (CMV) prophylaxis with ganciclovir/valganciclovir was administered to all high-risk recipients (CMV-positive donor/CMV IgG-negative recipients) and patients receiving anti-thymocyte globulin.

After skin preparation was performed in the operating room, the bladder was irrigated with 700 ml of normal saline. Thereafter, the bladder was filled with kanamycin solution and the urethral catheter was clamped until ureterovesical anastomosis was established. In most cases, the urethral catheters were removed on the third postoperative day. A double-J ureteral stent were inserted only in cases with complicated ureteral or bladder anatomy.

Based on our standard follow-up protocol, the renal allograft recipients were discharged at 10–15 days postoperatively, and they were followed on an outpatient basis once every week during the first month, once every other week until the third month, once a month until the first 2 years, and once every 2–3 months thereafter. Urinary cultures were performed when the patients had UTI symptoms or when the patients had newly detected pyuria on routine urinalysis examinations, which were performed at every visit. Patients were educated on the need for visiting the clinic as soon as the UTI symptoms were observed.

### Statistical analyses

The demographics of the study population were analyzed using descriptive statistics. Categorical data are presented as frequencies and percentages, whereas continuous variables are presented as means or medians, standard deviations, or interquartile ranges.

The relationship between the patient factors including perioperative colonization status and the development of early UTI was tested using chi-squared (χ^2^) test or Fisher’s exact test for dichotomous variables, and independent sample *t*-test or a Mann-Whitney *U* test for continuous variables. Factors with *P*<0.10 were then entered into a multivariate analysis model with binary logistic regression. To examine whether the perioperative colonization by uropathogens or early UTI were risk factors for graft outcomes (acute cellular rejection [ACR] and 5-year graft survival), univariate and multivariate analyses with Cox regression were used. To only include the ACRs and graft failure that developed after UTI, we used early UTI as a time-dependent covariate when assessing its relationship with ACR and graft failure. Statistical tests were performed using SPSS, version 19.0 (SPSS Inc., Chicago, IL), and a *P* value of <0.05 was considered statistically significant.

## Results

Among 450 patients who had received renal transplantation during the study period, 420 patients were included in the study. Thirty patients were excluded because of simultaneous transplantation of the other solid organs (liver, 5 patients; heart, 1 patient; pancreas, 14 patients), renal graft survival for <2 weeks (3 patients), and follow-up period of < 5 years (7 patients). The baseline characteristics of the study cohort are shown in Table 1.

**Table 1:** Baseline demographics and clinical characteristics (n = 420).

Female recipient	186 (44.3%)
Recipient age, years	40.5 (24–51)
Comorbidity	
Diabetes	86 (20.5%)
Hypertension	316 (75.2%)
Dialysis before transplantation	
No dialysis	53 (12.6%)
Hemodialysis	233 (55.5%)
Peritoneal dialysis	134 (12.9%)
Previous dialysis duration, days	683.5 (124–1908.5)
Vesicoureteral reflux	
No	211 (50.2%)
Yes	67 (16.0%)
Not examined	142 (33.8%)
Genitourinary abnormality	16 (3.8%)
Etiology of renal failure	
Hypertension	62 (14.8%)
IgA nephropathy	53 (12.6%)
Glomerulonephritis	50 (11.9%)
Diabetes	43 (10.2%)
Polycystic kidney	23 (5.5%)
Focal segmental glomerulosclerosis	22 (5.2%)
Reflux nephropathy	19 (4.5%)
Others	43 (10.2%)
Unknown	105 (25.0%)
Transplantation characteristics	
Female donor	179 (42.6%)
Donor age, years	41 (31–49)
Deceased donor transplantation	155 (36.9%)
Second transplantation	25 (6.0%)
Double-J ureteral stent insertion	11 (2.6%)
Prolonged use of urinary catheter^a^	47 (11.2%)
Antibiotic prophylaxis^b^	17 (4.0%)
Delayed graft function	8 (1.9%)
Immunosuppressant regimen	
Induction therapy	
None	135 (32.1%)
Basiliximab	284 (67.6%)
Anti-thymocyte globulin	1 (0.2%)
Maintenance therapy	
Tacrolimus-based	354 (85.1%)
Cyclosporine A-based	62 (14.9%)
Five-year outcome	
≥1 acute cellular rejection	179 (42.6%)
5-year renal graft failure	12 (2.9%)
5-year mortality	7 (1.7%)

Data reported as median (IQR) or n (%).

^*a*^Urinary catheter use for >4 days

^*b*^3-month prophylaxis with trimethoprim-sulfomethoxazole

### Bacterial species isolated from perioperative genitourinary specimens

**Fig 1:**
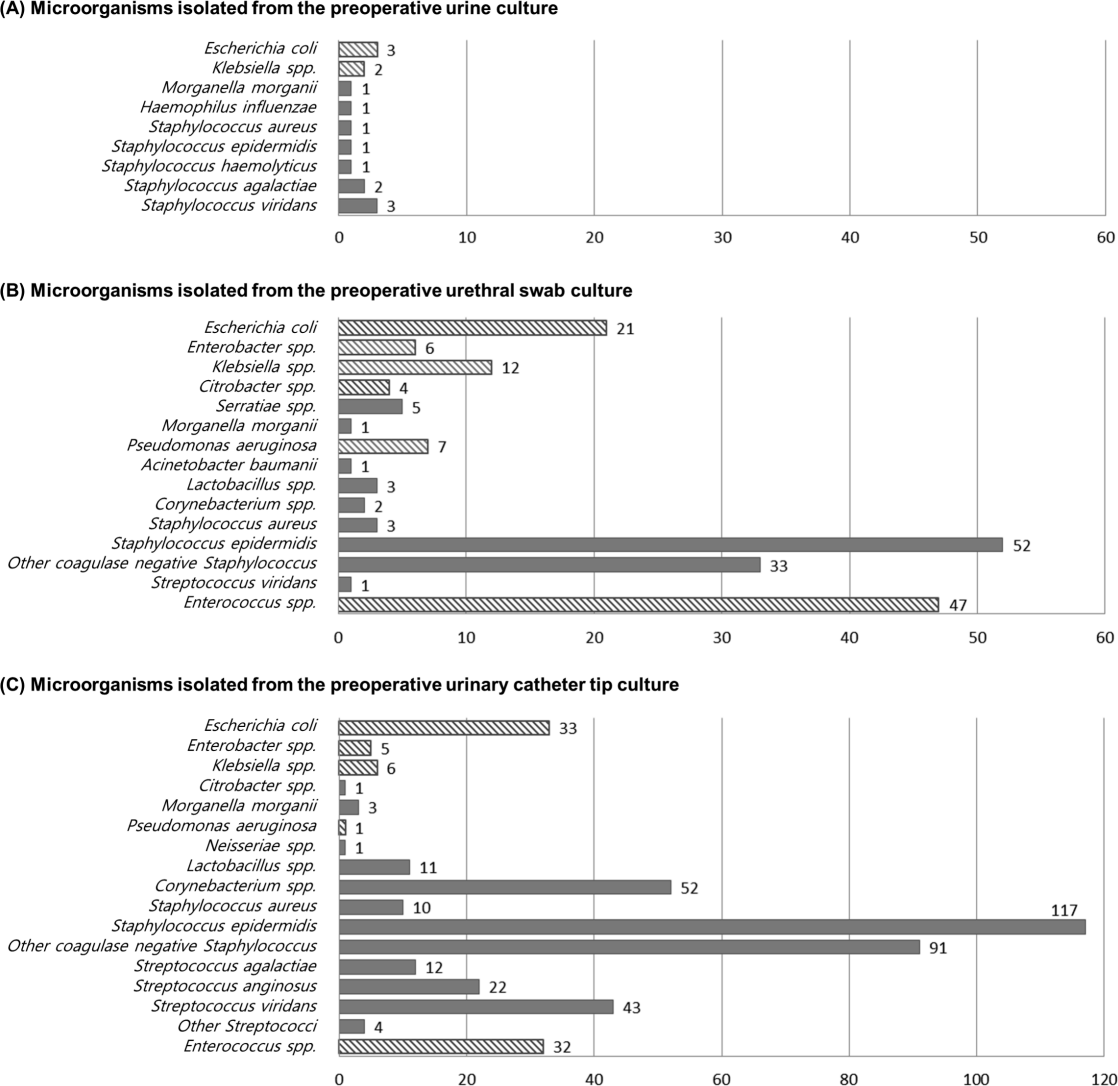
Organisms isolated from perioperative surveillance cultures of renal transplant recipients. Microorganisms isolated from preoperative urinary (A), preoperative urethral swab (B), and postoperative urinary catheter tip specimens (C). The shaded box depicts the common uropathogens.

Microbiologic culture specimens, including preoperative urine, preoperative urethral swabs, and postoperative urinary catheter tips, were available in 419 (99.7%), 381 (90.7%), and 385 (91.6%) patients, respectively. The culture results were positive in only 15 (3.6%) of the preoperative urine samples and all indicated a single microorganism (Fig 1A). Among the preoperative urethral swab cultures, 311 (81.6%) indicated positive results for bacterial growth, and 39.5% of these indicated >2 microorganisms (mean, 1.5 microorganism per positive specimen). The common microorganisms isolated included those known to comprise skin flora, such as coagulase-negative *Staphylococcus* and *Corynebacterium* species, followed by those of the *Enterobacteriaceae* family, *E*. *coli*, and *Enterococcus* species (Fig 1B). A total of 160 (41.5%) urinary catheter tips that were cultured during removal indicated microorganism growth; among these, 39 (24.4%) indicated polymicrobial growth (Fig 1C). The microorganisms frequently isolated from the tips were similar to those isolated from urethral swab specimens, except for a reduced proportion of *Corynebacterium* species. Uropathogens—bacteria commonly associated with UTIs, including *E*.*coli*, *Klebsiella* species, *Enterobacter* species, *Pseudomonas aeruginosa*, and *Enterococcus* species—were observed in 8 (1.9%) preoperative urinary specimens, 68 (17.8%) preoperative urethral swab specimens, and 73 (19.0%) postoperative urinary catheter tip specimens. Overall, uropathogenic bacteria were grown from ≥1 perioperative genitourinary specimens from 132 (31.4%) patients.

### Incidence of early UTI and causative organisms isolated during the first UTI episode

During the 6 months after renal transplantation, 26 patients (6.2%) had ≥1 episode of microbiologically confirmed bacterial UTI. Five (19.2%) of the 26 patients presented with lower UTI symptoms, and 21 (80.8%) presented with acute pyelonephritis. Microorganisms that had grown in significant concentrations from the urine specimens during the first UTI episodes are shown in Table 2. *E*. *coli* was the most commonly isolated organism, followed by *Klebsiella* species and *Enterococcus* species. Antimicrobial susceptibility tests indicated that 5 isolates (19.2%) were MDR, including 2 *E*. *coli*, 2 *Klebsiella pneumoniae*, and 1 *Enterobacter cloacae* isolate. The antimicrobial susceptibility patterns of the isolated microbial agents are shown in S1 Table.

**Table 2:** Thirty-eight causative microorganisms isolated from urine during the first early urinary tract infection episodes in 37 cases after renal transplantation.

Microorganisms		Number of positive isolates (n = 26)	(%)
Gram-negative rods	*Escherichia coli*^a^	9	34.6
	*Klebsiella spp*.^a^^,^^b^	6	23.1
	*Pseudomonas aeruginosa*^a^	3	7.7
	*Enterobacter spp*.^a^	2	7.7
	*Sphingomonas paucimobilis*	1	3.8
Gram-positive cocci	*Enterococcus spp*.^a^^,^^c^	4	19.2
	*Staphylococcus epidermidis*	1	3.8

^*a*^Common uropathogens

^*b*^*Klebsiella pneumonia* (n = 5), *Klebsiella oxytoca* (n = 1)

^*c*^*Enterococcus faecalis* (n = 3), *Enterococcus faecium* (n = 1)

### Association between prior genitourinary colonization by uropathogens and postoperative early UTI

Of the 132 renal transplant recipients with perioperative colonization with common uropathogens, 13 (9.8%) developed early UTI, in contrast with 13 (4.5%) of 288 patients who were previously free of uropathogens but subsequently developed early UTI. The presence of uropathogens in perioperative genitourinary specimens was positively associated with an increased risk of early UTI (odds ratio [OR], 3.23; 95% confidence interval [CI], 1.44 to 7.24; *P* = 0.003) (Table 3). Other factors that were significantly associated with early UTI included female gender, and dialysis for >1 year before transplantation. In multivariate analysis, the perioperative presence of an uropathogen remained an independent factor associated with early UTI (OR, 3.33; 95% CI, 1.46 to 7.59; *P* = 0.004), along with second transplantation (OR, 3.42; 95% CI, 1.04 to 11.21; *P* = 0.04), and delayed graft function (OR 7.20; 95% CI, 1.29 to 40.30; *P* = 0.03) (Table 3).

**Table 3:** Factors associated with early urinary tract infection.

	Total	Early UTI	Univariate analysis		Multivariate analysis^a^	
	n (%)	n (%)	OR (95% CI)	*P* value	OR (95% CI)	*P* value
Female recipient	186 (44.3%)	17 (65.4%)	2.51 (1.01–5.78)	0.03		
Recipient age, years	40.5 (24.0–51.0)	36.5 (17.8–52.8)		0.60		
Diabetes	86 (20.5%)	4 (15.4%)	0.69 (0.23–2.06)	0.51		
Hypertension	316 (75.2%)	18 (5.7%)	0.73 (0.31–1.72)	0.46		
Vesicoureteral reflux^b^	67 (24.1%)	3 (23.1%)	0.94 (0.25–3.53)	1.00		
Genitourinary abnormality	16 (3.8%)	3 (11.5%)	3.82 (1.02–14.37)	0.07		
Previous dialysis	367 (87.4%)	24 (92.3%)	1.78 (0.41–7.78)	0.76		
Dialysis for >1 year	265 (63.1%)	21 (80.8%)	2.58 (0.95–6.99)	0.05		
Anuria before TPL^c^	96 (23.6%)	9 (34.6%)	1.78 (0.77–4.14)	0.17		
Female donor	179 (42.7%)	11 (42.3%)	0.98 (0.44–2.19)	0.97		
Donor age, years	41.0 (31.0–49.0)	42.0 (36.5–50.5)		0.35		
Deceased donor TPL	155 (36.9%)	12 (46.2%)	0.66 (0.30–1.47)	0.31		
Second TPL	25 (6.0%)	4 (15.4%)	3.23 (1.02–10.22)	0.06	3.42 (1.04–11.21)	0.04
Double-J ureteral stent insertion	11 (2.6%)	2 (7.7%)	3.57 (0.73–17.4)	0.14		
Prolonged use of urinary catheter^d^	47 (11.2%)	5 (19.2%)	2.00 (0.72–5.57)	0.19		
Antibiotic prophylaxis	17 (4.0%)	1 (38%)	1.01 (0.13–7.96)	0.15		
Cyclosporine-based maintenance therapy	62 (14.8%)	5 (19.2%)	1.41 (0.51–3.88)	0.57		
Delayed graft function	8 (1.9%)	2 (7.7%)	5.39 (1.03–28.13)	0.08	7.20 (1.29–40.30)	0.03
Uropathogen isolated perioperatively	132 (31.4%)	15 (57.7%)	3.23 (1.44–7.24)	0.003	3.33 (1.46–7.59)	0.004
From preoperative urine	8 (1.9%)	2 (7.7%)	5.39 (1.03–28.13)	0.08		
From urethral swab	68 (16.2%)	9 (34.6%)	3.01 (1.28–7.06)	0.02		
From urinary catheter tip	73 (17.4%)	10 (38.5%)	3.28 (1.43–7.57)	0.007		

UTI, urinary tract infection; TPL, transplantation; HR, hazard ratio; CI, confidence interval.

^*a*^Factors with p<0.10 in univariate analysis (female sex, second transplantation, genitourinary abnormality, previous dialysis >1year, delayed graft function, and isolation of uropathogens perioperatively) were included in the multivariate logistic regression (backward LR).

^*b*^Vesicoureteral reflux of the native kidney on voiding cystourethrography conducted preoperatively

^*c*^Anuria before TPL was defined as less than 200ml urine per day in patients who were on dialysis for more than 3 years. Values were missing in 14 patients.

^*d*^Urinary catheter use for >4 days

### Comparison between causative organism of early UTI and prior perioperative genitourinary culture results

We reviewed the perioperative genitourinary culture results of 26 patients with early UTI, and compared these findings with the causative microorganisms isolated during the early UTI episode. Microorganisms that were identical in terms of species and antibiotic susceptibility profile were observed in 9 (34.6%) patients. The specimens from which the identical organisms had been isolated varied, and in some cases, the organisms were identified in >1 specimen (4 from urinary catheter tip cultures, 3 from urethral cultures, 1 from urethral and urinary catheter cultures, and 1 from urinary and urethral swab cultures).

However, in the remaining 17 (65.4%) patients, the culture results did not match. In 15 patients, ≥1 organisms were isolated from perioperative cultures, but were not identical to the causative organisms identified during postoperative UTI; in 2 patients, all the perioperative specimens were sterile. In particular, in 5 (19.2%) patients, the previous culture results were misleading as the previous perioperative specimens had grown uropathogens that were not the causative organism of the subsequent UTI. Also in 5 patients, different uropathogens were isolated from two different specimen, further limiting its ability to predict the causative organism.

### Association between prior genitourinary colonization by uropathogens, postoperative early UTI, and graft outcome

We further examined whether perioperative genitourinary colonization by uropathogens or early UTI was associated with acute rejection and 5-year graft survival. Although early UTI was associated with graft loss at 5 years in univariate Cox regression analysis (HR, 5.48; 95% CI, 1.48 to 20.25; *P* = 0.01) it was not a significant factor in the multivariate analysis (Table 4). Neither colonization by uropathogens nor early UTI was significantly associated with ACR (S2 Table).

**Table 4:** Factors associated with 5-year graft survival.

	Total n (%)	Graft failure n (%)	Univariate analysis		Multivariate analysis^a^	
			HR (95% CI)	*P* value	HR (95% CI)	*P* value
Female recipient	186 (44.3%)	5 (41.7%)	0.90 (0.29–2.83)	0.85		
Recipient age, years	40.5 (24.0–51.0)	26.5 (17.8–42.5)	0.97 (0.94–1.00)	0.09		
Diabetes	86 (20.5%)	4 (33.3%)	2.06 (0.62–6.85)	0.24		
Hypertension	316 (75.2%)	9 (75.0%)	0.99 (0.27–3.65)	0.98		
Vesicourethral reflux^b^	67 (24.1%)	2 (22.2%)	0.94 (0.19–4.50)	0.93		
Genitourinary abnormality	16 (3.8%)	0 (0%)	–	0.64		
Previous dialysis	368 (87.6%)	11 (91.7%)	1.53 (0.20–11.87)	0.68		
Dialysis for >1 year	265 (63.1%)	10 (83.3%)	3.02 (0.66–13.80)	0.15		
Anuria before TPL^c^	96 (23.6%)	7 (58.3%)	4.69 (1.49–14.78)	0.008		
Female donor	179 (42.7%)	5 (41.7%)	0.93 (0.30–2.97)	0.92		
Donor age >60 years	21 (5.0%)	0	–	0.61		
Deceased donor TPL	155 (36.9%)	6 (50.0%)	0.57 (0.18–1.76)	0.33		
Second TPL	25 (6.0%)	3 (25.0%)	5.46 (1.48–20.17)	0.01	6.81 (1.84–25.2)	0.004
Number of HLA mismatches				0.99		
0	34 (8.1%)	1 (8.3%)				
1	19 (4.5%)	1 (8.3%)	1.72 (0.11–27.45)	0.70		
2	53 (12.6%)	1 (8.3%)	0.61 (0.04–9.80)	0.73		
3	129 (30.7%)	4 (33.3%)	1.03 (0.12–9.18)	0.98		
4	92 (21.9%)	3 (25.0%)	1.08 (0.11–10.36)	0.95		
5	72 (17.1%)	2 (16.7%)	0.94 (0.09–10.37)	0.96		
6	21 (5.0%)	0	–	0.98		
Double–J ureteral stent insertion	11 (2.6%)	1 (8.3%)	3.67 (0.47–28.40)	0.21		
Prolonged use of urinary catheter^d^	47 (11.2%)	2 (16.7%)	1.61 (0.35–7.34)	0.54		
Antibiotic prophylaxis	17 (4.0%)	2 (16.7%)	4.88 (1.07–22.26)	0.04		
Induction agent				0.87		
None	135 (32.1%)	3 (25.0%)				
Basiliximab	284 (67.6%)	9 (75.0%)	1.42 (0.39–5.25)	0.60		
Anti–thymocyte globulin	1 (0.2%)	0	–	0.99		
Cyclosporine-based maintenance therapy	62 (14.8%)	2 (16.7%)	1.15 (0.25–5.24)	0.86		
Delayed graft function	8 (1.9%)	0	0.05 (0 to <0.01)	0.76		
Acute rejection	179 (42.6%)	11 (91.7%)	15.13 (1.95–117.19)	0.009	16.17 (2.08–125.45)	0.008
Uropathogen isolated perioperatively	132 (31.4%)	6 (50.0%)	2.22 (0.72–6.90)	0.17		
Early UTI	26 (6.2%)	3 (25.0%)	5.48 (1.48–20.25)	0.01		
CMV infection	12 (2.9%)	1 (8.3%)	3.03 (0.39–23.50)	0.29		
BK virus infection	45 (10.7%)	2 (16.7%)	1.62 (0.36–7.41)	0.53		

HR, hazard ratio; CI, confidence interval; TPL, transplantation,; HLA, human leukocyte antigen; UTI, urinary tract infection; CMV, cytomegalovirus.

^*a*^Multivariate Cox regression (Backward LR) analysis performed with 406 cases which had no missing data for all included variables.

^*b*^Vesicoureteral reflux of native kidney on voiding cystourethrography preoperatively

^*c*^Anuria before TPL was defined as less than 200ml urine per day in patients who were on dialysis for more than 3 years. Values were missing in 14 patients.

^*d*^Urinary catheter use for more than 4 days

## Discussion

Considering the growing increase in the number of pathogens with antimicrobial resistance, the accurate and prompt diagnosis of causative microorganisms is becoming more important. In the present study, we aimed to assess whether the results of perioperative genitourinary specimen cultures would help predict the causative organisms of early UTI, and could thus be used to guide the initial selection of antimicrobials. According to our data, the usefulness of the results of previous perioperative genitourinary specimen cultures in predicting the exact causative organism during the subsequent early UTI episodes appears to be limited, given the low concordance rate. Although the colonization of the vaginal and urethral mucosa has been known to precede bacterial UTI [21, 22], and early infection after transplantation is known to be partly caused by the previously colonized organisms in the recipients [19], our results suggest that, in at least 65% of the patients, uropathogens other than those present during the perioperative period were responsible for the UTI, even in the early periods after transplantation. This may reflect the dynamic nature of the genitourinary microflora, which involve constant challenges by different uropathogens.

The overall prevalence of UTI in the present study (6.2%) was lower than that reported in most of the previous studies [2], even though we only considered the first UTI event within the 6-month postoperative period. This may be explained by the difference in the UTI definitions used across the studies. In most studies, patients underwent routine screening culture for bacteriuria during every outpatient visit and asymptomatic bacteriuria was considered as a UTI. However, the clinical value of screening and treating cases of asymptomatic bacteriuria are controversial [23, 24]. In the present study, only symptomatic bacteriuria was considered as UTI for clinical relevance. The common uropathogens comprised 86.8% of the causative bacteria isolated during the UTI episode.

The rate of MDR organisms was lower than that in other recent reports (19.2% vs. 40–70%) [25–27], however the fact that 26% of the common uropathogens isolated during the early UTI episode were resistant to our initial choice of empiric therapy—ciprofloxacin—was alarming (S1 Table). While this calls for an improvement in the choice of initial empirical antibiotics, our results suggest that the information about the perioperative colonized organism and its resistant pattern would have little additional value in the selection of the empirical therapy, as it may falsely direct physicians in some cases. We also performed additional analysis, however were unable to characterize any subgroups among the early UTI patients who were more likely to have UTIs caused by colonized organisms (data not shown). Identification of such subgroup would be valuable in early refining of antimicrobials. On the other hand, undoubtedly, the best approach to select targeted antimicrobial would be early identification of the causative organism itself. Recently, rapid diagnostic methods based on technologies such as matrix-assisted laser desorption ionization-time of flight mass spectrometry, nucleic acid amplification techniques, next generation-sequencing, and biosensors, are being used to identify pathogens and provide resistance information within hours in the research setting; we believe that the application of these methods will shorten the time to targeted therapy [28].

Although only 11.3% of patients colonized with uropathogens subsequently developed early UTI, perioperative colonization with uropathogens was found to be significantly associated with a higher risk of early UTI, along with second transplantation, and delayed graft function. Considering the low concordance rate between the perioperatively colonized organism and the causative organism of the early UTI episode, this relationship is likely to be indirect. Certain behavioral or environmental factors of the host may have rendered patients more prone to both perioperative colonization and postoperative infection by uropathogens. Regardless of the nature of the relationship, knowledge of the perioperative colonization status may be useful in identifying patients at high risk of early UTI, particularly when combined with the other risk factors of early UTI.

When we further evaluated the impact of perioperative colonization and early UTI on the development of ACR and 5-year graft survival, no significant association was observed. Previous studies have reported divergent findings in terms of the impact of UTI on renal allograft function and survival, which can be partly explained by the period during which the infection occurred and its severity. With regard to the early period, Lee et al. reported that untreated UTI after transplantation was associated with an increased risk of ACR [29], whereas Giral et al. reported that acute graft pyelonephritis was associated with inferior graft survival [5]. The negative results of the present study, in comparison with the report of Lee et al., may have been influenced by the differences in the definition of UTI. The results of the study by Lee et al. may instead reflect the relationship between untreated asymptomatic UTI and graft outcome, as cases of bacteriuria without any symptoms were considered as UTI cases in that study and most of the untreated UTI cases were asymptomatic.

Our study is unique in that we examined the perioperative genitourinary colonization status and explored its relationship with early UTI in renal transplant patients. The notable strength of this study is that it is based on data from systematically collected genitourinary specimens from a relatively large number of patients.

The present study has several limitations. First, this is a retrospective study conducted in a single transplantation center. As genitourinary colonizers, common uropathogens, and their antibiotic susceptibility vary based on the time and region, the bacterial profile in the present study may not be widely generalizable. Moreover, the fact that postoperative TMP-SMX prophylaxis was not routine practice in our center during the study period should also be considered. Whether TMP-SMX prophylaxis influences the UTI rate or the resistance pattern of causative organisms is currently controversial. Although systemic review published in 2011 had concluded that the TMP-SMX prophylaxis lowered the risk of bacteriura, a recent before- after study by Singh et al., showed that TMP-SMX prophylaxis had no impact on the prevalence of asymptomatic bacteriuria or UTI, but was rather associated with an increase in TMP-SMX and amoxicillin resistance among bacterial isolates [30, 31]. There was no significant association between TMP/SMX prophylaxis and rate of early UTI in our study, however it is hard to draw a meaningful conclusion as TMP-SMX was administered in only 4%. Nevertheless, the low rate of TMP-SMX use may have contributed to the low rate of MDR organisms of our study. Finally, as we used the classic culture-based method, rather than genomic analysis, to identify and characterize the colonized organisms, the differentiation of strains with high pathogenic potential and low-risk commensal strains was not possible. Hence, patients with commensal strains of species known to be common uropathogens may have been incorrectly included as uropathogen colonizers, which may have contributed to the low predictability of future UTI development. Also without genomic typing, we were unable to differentiate between various strains, and this may have falsely increased the concordance rate.

## Conclusions

Renal transplant recipients who were colonized with common uropathogens perioperatively were more likely to develop early UTI. However, the concordance rate between the culture results of the perioperative colonizing organisms and the actual causal pathogen during the UTI event was low, indicating that the usefulness of the culture results of perioperative colonizers in guiding the initial antimicrobial therapy during early UTI may be limited.

## Supporting information

S1 Table Antimicrobial susceptibility profile of frequent urinary isolates during early urinary tract infection. (DOCX)

S2 Table Factors associated with biopsy-proven acute cellular rejection. (DOCX)
