# Supplementary material for: Value of perioperative genitourinary screening culture and colonization status in predicting early urinary tract infection after renal transplantation
Source: PLoS One. 2018 Apr 19;13(4):e0196115. doi: 10.1371/journal.pone.0196115 (PMC5908147; doi:10.1371/journal.pone.0196115)
Supplement: S1 Table — (DOCX) [file pone.0196115.s001.docx]

**S1 Table. Antimicrobial susceptibility profile of frequent urinary isolates during early urinary tract infection**

|  | N_total_ | Ampicillin | Cefotaxime | Ceftazidime | Imipenem | Ciprofloxacin | PIP/Tazo | TMP/SMX | Gentamycin | Vancomycin |
| --- | --- | --- | --- | --- | --- | --- | --- | --- | --- | --- |
| *Escherichia coli* | 9 | 4/9 | 9/9 | 8/9 | 9/9 | 7/9 | 9/9 | 6/7 | 6/9 | R |
|  |  | (44.4%) | (100%) | (88.9%) | (100%) | (77.8%) | (100%) | (85.7%) | (66.7%) |  |
| Klebsiella species | 6 | R | 3/6 | 4/6 | 6/6 | 3/6 | 6/6 | 4/4 | 4/6 | R |
|  |  |  | (50.0%) | (66.7%) | (100%) | (50.0%) | (100%) | (100%) | (66.7%) |  |
| Enterococcus species | 4 | 1/4 | NT | NT | NT | 2/3 | NT | R | 2/4 | 4/4 |
|  |  | (25.0%) |  |  |  | (66.7%) |  |  | (50.0%) | (100%) |
| Pseudomonas species | 3 | R | R | 3/3 | 3/3 | 3/3 | 3/3 | R | 3/3 | R |
|  |  |  |  | (100%) | (100%) | (100%) | (100%) |  | (100%) |  |
| Enterobacter species | 2 | R | 1/2 | 1/2 | 2/2 | 1/2 | 2/2 | 0/1 | 2/2 | R |
|  |  |  | (50.0%) | (50.0%) | (100%) | (50.0%) | (100%) | (0%) | (100%) |  |

Data are presented as the number of sensitive isolates/number of isolates tested (%), unless otherwise indicated. PIP/Tazo, piperacillin/tazobactam; TMP/SMX, trimethoprim/sulfamethoxazole; R, bacteria inherently resistant to the selected antimicrobial; NT, not tested.

^1^sensitive to high-level gentamycin
