# Supplementary material for: Value of perioperative genitourinary screening culture and colonization status in predicting early urinary tract infection after renal transplantation
Source: PLoS One. 2018 Apr 19;13(4):e0196115. doi: 10.1371/journal.pone.0196115 (PMC5908147; doi:10.1371/journal.pone.0196115)
Supplement: S2 Table — (DOCX) [file pone.0196115.s002.docx]

**S2 Table. Factors associated with biopsy-proven acute cellular rejection**

|  | Total n (%) | Acute cellular rejection (%) | Univariate analysis | | Multivariate analysis^1^ | |
| --- | --- | --- | --- | --- | --- | --- |
|  |  |  | HR (95% CI) | *P* value | HR (95% CI) | *P* value |
| Female recipient | 186 (44.3%) | 72 (40.2%) | 0.83 (0.62–1.12) | 0.23 |  |  |
| Recipient age, years | 40.5 (24.0–51.0) | 39.0 (28.0–51.0) | 1.00 (0.99–1.01) | 0.92 |  |  |
| Diabetes | 86 (20.5%) | 37 (20.7%) | 0.05 (0.73–1.51) | 0.78 |  |  |
| Hypertension | 316 (75.2%) | 139 (77.7%) | 1.19 (0.84–1.69) | 0.34 |  |  |
| Vesicourethral reflux^2^ | 67 (24.1%) | 28 (23.7%) | 1.01 (0.66–1.55) | 0.96 |  |  |
| Genitourinary abnormality | 16 (3.8%) | 7 (3.9%) | 1.00 (0.47–2.13) | 0.10 |  |  |
| Previous dialysis | 368 (87.6%) | 158 (88.3%) | 1.07 (0.68–1.68) | 0.79 |  |  |
| Dialysis for >1 year | 265 (63.1%) | 125 (69.8%) | 1.58 (1.15–2.18) | 0.005 |  |  |
| Anuria before TPL^3^ | 96 (23.6%) | 55 (31.8%) | 1.77 (1.29–2.45) | <0.001 |  |  |
| Female donor | 179 (42.7%) | 85 (47.5%) | 1.26 (0.94–1.69) | 0.12 |  |  |
| Donor age >60 years | 21 (5.0%) | 15 (8.4%) | 2.85 (1.68–4.85) | <0.001 | **1.83 (1.05–3.19)** | **0.03** |
| Deceased donor transplantation | 155 (36.9%) | 89 (49.7%) | 2.18 (1.62–2.93) | <0.001 | **2.16 (1.54–3.03)** | **<0.001** |
| Second transplantation | 25 (6.0%) | 11 (6.1%) | 1.08 (0.59–1.99) | 0.81 |  |  |
| Number of HLA mismatches |  |  |  | <0.001 |  | **0.02** |
| 0 | 34 (8.1%) | 8 (4.5%) |  |  |  |  |
| 1 | 19 (4.5%) | 3 (1.7%) | 0.69 (0.18–2.62) | 0.59 | 0.77 (0.20–2.89) | 0.70 |
| 2 | 53 (12.6%) | 17 (9.5%) | 1.50 (0.65–3.48) | 0.34 | 1.98 (0.85–4.62) | 0.11 |
| 3 | 129 (30.7%) | 52 (40.3%) | 1.99 (0.95–4.20) | 0.07 | 2.34 (1.11–4.95) | 0.03 |
| 4 | 92 (21.9%) | 46 (25.7%) | 2.70 (1.27–5.72) | 0.01 | 2.06 (0.97–4.41) | 0.06 |
| 5 | 72 (17.1%) | 40 (22.3%) | 3.49 (1.63–7.47) | 0.001 | 2.82 (1.31–6.08) | 0.008 |
| 6 | 21 (5.0%) | 13 (7.3%) | 4.08 (1.69–9.84) | 0.002 | 3.84 (1.57–9.41) | 0.003 |
| Double-J ureteral stent insertion | 11 (2.6%) | 3 (1.7%) | 0.54 (0.17–1.70) | 0.30 |  |  |
| Prolonged use of urinary catheter^4^ | 47 (11.2%) | 15 (8.4%) | 0.67 (0.39–1.14) | 0.14 |  |  |
| Antibiotic prophylaxis | 17 (4.0%) | 11 (6.1%) | 2.06 (1.12–3.79) | 0.02 | **2.20 (1.15–4.21)** | **0.02** |
| Induction agent |  |  |  | 0.002 |  |  |
| None | 135 (32.1%) | 42 (23.5%) |  |  |  |  |
| Basiliximab | 284 (67.6%) | 137 (76.5%) | 1.87 (1.32–2.64) | <0.001 |  |  |
| Anti-thymocyte globulin | 1 (0.2%) | 0 (0%) | 0 | 0.96 |  |  |
| Cyclosporine-based maintenance therapy | 62 (14.8%) | 15 (8.4%) | 0.45 (0.26–0.76) | 0.003 |  |  |
| Delayed graft function | 8 (1.9%) | 2 (1.1%) | 0.57 (0.14–2.28) | 0.42 |  |  |
| Uropathogen isolated perioperatively | 132 (31.4%) | 49 (27.4%) | 0.77 (0.55–1.07) | 0.11 |  |  |
| Early UTI (time-dependent) | 26 (6.2%) | 13 (7.3%) | 1.627 (0.76–3.50) | 0.21 |  |  |
| CMV infection | 12 (2.9%) | 5 (2.8%) | 0.96 (0.39–2.33) | 0.92 |  |  |
| BK virus infection | 45 (10.7%) | 27 (15.1%) | 1.62 (1.07–2.44) | 0.02 |  |  |

HR, hazard ratio; CI, confidence interval; HLA, Human leukocyte antigen; UTI, urinary tract infection; CMV, cytomegalovirus.

^1^Multivariate Cox regression (backward LR) analysis was performed with 406 cases that had no missing data for all the included variables.

^2^Vesicoureteral reflux of the native kidney on voiding cystourethrography, conducted preoperatively

^3^Anuria before TPL was defined as less than 200cc urine per day in patients who were on dialysis for more than 3 years. Values were missing in 14 patients.

^4^Urinary catheter use for more than 4 days
